# Radiogenomic System for Non-Invasive Identification of Multiple Actionable Mutations and PD-L1 Expression in Non-Small Cell Lung Cancer Based on CT Images

**DOI:** 10.3390/cancers14194823

**Published:** 2022-10-02

**Authors:** Jun Shao, Jiechao Ma, Shu Zhang, Jingwei Li, Hesen Dai, Shufan Liang, Yizhou Yu, Weimin Li, Chengdi Wang

**Affiliations:** 1 Department of Respiratory and Critical Care Medicine, Med-X Center for Manufacturing, West China Hospital, West China School of Medicine, Sichuan University, No. 37 GuoXue Alley, Chengdu 610041, China; 2 AI Lab, Deepwise Healthcare, No. 8 Haidian Street, Beijing 100080, China; 3 Department of Computer Science, The University of Hong Kong, Pokfulam, Hong Kong 999077, China

**Keywords:** actionable mutations, non-small cell lung cancer, deep learning, radiomics, molecular status

## Abstract

**Simple Summary:**

Actional mutations and PD-L1 expression are of paramount importance for the precision treatment of lung cancer. Radiogenomics is a promising field that integrated radiologic images and genomic data through artificial intelligence technology. This approach enables non-invasive assessment of genes, but the vast majority of studies are limited to single gene mutation prediction. Our study aimed to propose a multi-label multi-task deep learning (MMDL) system to predict molecular status based on routinely acquired computed tomography (CT) images using deep learning and radiomics. A dataset of CT images from 1096 non-small cell lung cancer (NSCLC) patients with molecular tests was curated to train, validate and test. The MMDL model achieved superior performance on the classification task of simultaneous identification of eight genes or even ten molecules. This system has the potential to be an auxiliary support tool to advance precision oncology.

**Abstract:**

Purpose: Personalized treatments such as targeted therapy and immunotherapy have revolutionized the predominantly therapeutic paradigm for non-small cell lung cancer (NSCLC). However, these treatment decisions require the determination of targetable genomic and molecular alterations through invasive genetic or immunohistochemistry (IHC) tests. Numerous previous studies have demonstrated that artificial intelligence can accurately predict the single-gene status of tumors based on radiologic imaging, but few studies have achieved the simultaneous evaluation of multiple genes to reflect more realistic clinical scenarios. Methods: We proposed a multi-label multi-task deep learning (MMDL) system for non-invasively predicting actionable NSCLC mutations and PD-L1 expression utilizing routinely acquired computed tomography (CT) images. This radiogenomic system integrated transformer-based deep learning features and radiomic features of CT volumes from 1096 NSCLC patients based on next-generation sequencing (NGS) and IHC tests. Results: For each task cohort, we randomly split the corresponding dataset into training (80%), validation (10%), and testing (10%) subsets. The area under the receiver operating characteristic curves (AUCs) of the MMDL system achieved 0.862 (95% confidence interval (CI), 0.758–0.969) for discrimination of a panel of 8 mutated genes, including *EGFR*, *ALK*, *ERBB2*, *BRAF*, *MET*, *ROS1*, *RET* and *KRAS*, 0.856 (95% CI, 0.663–0.948) for identification of a 10-molecular status panel (previous 8 genes plus *TP53* and PD-L1); and 0.868 (95% CI, 0.641–0.972) for classifying *EGFR* / PD-L1 subtype, respectively. Conclusions: To the best of our knowledge, this study is the first deep learning system to simultaneously analyze 10 molecular expressions, which might be utilized as an assistive tool in conjunction with or in lieu of ancillary testing to support precision treatment options.

## 1. Introduction

Lung cancer is the malignancy tumor with the highest mortality worldwide driven by multiple genetic mutations, approximately 85% of which is non-small cell lung cancer (NSCLC) [1,2]. Personalized treatments of patients with NSCLC, such as targeted therapy and immunotherapy, have shifted the paradigm that relies on the exact molecular profile [3]. The National Comprehensive Cancer Network (NCCN) guidelines recommend that the statuses of several genomic alterations should be identified for appropriate drug selection, including epidermal growth factor receptor (*EGFR*), anaplastic lymphoma receptor tyrosine kinase (*ALK*), erb-b2 receptor tyrosine kinase 2 (*ERBB2*), V-Raf murine sarcoma viral oncogene homolog B1 (*BRAF*), mesenchymal-epithelial transition factor (*MET*), c-ROS proto-oncogene 1 (*ROS1*), rearranged during transfection (*RET*), and Kirsten rat sarcoma viral oncogene (*KRAS*) [4,5]. In addition, the mutation of tumor protein p53 (*TP53*) and tumor proportion score (TPS) of programmed death ligand-1 (PD-L1) are also closely related to lung cancer treatment decisions [6,7,8,9].

Traditionally, the detection of the above molecular alterations relied on quantitative polymerase chain reaction (qPCR), next-generation sequencing (NGS), or immunohistochemistry (IHC) [10,11]. Unfortunately, the majority of clinical institutions only perform sequential molecular testing on a single gene. While the NGS-based assays capable of detecting multiple genes are prohibitively expensive. The IHC requires a time-consuming visual inspection of histopathology slides by experienced pathologists. Moreover, these approaches depend on invasive biopsy or surgery to obtain tumor tissues [12,13]. The results are affected by insufficient tumor quantity or quality, as well as sample heterogeneity, hindering the widespread clinical application. Therefore, there is an urgent demand for a non-invasive and efficient genetic testing method.

Radiogenomic is a promising field that integrates radiologic images and genomic data through artificial intelligence (AI) technology. Initially, researchers observed a relationship between gene expression and quantitative imaging features. For instance, *ALK* rearrangement is associated with large pleural effusion, and *EGFR* mutations have been linked to irregular nodules [14,15,16,17]. Due to the development of computing power, high-throughput features are able to be captured from computed tomography (CT) images and handle complex tasks. Deep learning, a particular machine learning approach, has been widely applied in medical diagnosis tasks including skin cancer detection, COVID-19 diagnosis, and lung cancer screening [18,19,20,21,22,23,24]. Similarly, deep learning has been also utilized to build genetic prediction models based on image features. A deep learning model using whole-lung CT imaging has been constructed to evaluate *EGFR* status with an area under the receiver operating characteristic curve (AUC) of 0.748 to 0.813 in six testing cohorts [25]. However, previous studies have mostly assessed a single gene or two genes, ignoring the clinical need to assess multiple genes [26,27]. Other researchers extracted 1672 radiomic features from three-dimensional CT patches to simultaneously determine the presence of *EGFR*, *KRAS*, *ERBB2*, and *TP53* mutations, but the approach was developed with a small sample size of chest images from 134 NSCLC patients [28]. Hence, large-scale samples and multiple-molecules analyses are warranted for research.

**Figure 1:**
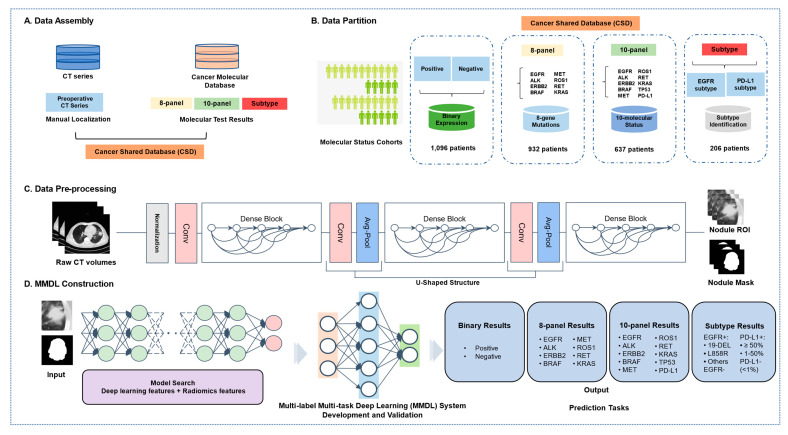
Overall workflow of the MMDL system. (**A**) Data assembly included the original CT image data, molecular status, subtypes, and clinical records. (**B**) Data partition: for model development and validation, the acquired data set was further partitioned into target binary expression (positive expression of at least one molecule and all negative molecular expression ), multiple molecular status prediction tasks (8-panel or 10-panel), and subtype prediction tasks (19-DEL, L858R, other mutation and wild type for *EGFR*; TPS cut-off of 50%: low PD-L1+ and high PD-L1+ for PD-L1 positive expression, and PD-L1- represents the negative expression of PD-L1). (**C**) The DenseNet backbone-based U-Shaped deep learning segmentation architecture performed tumor mask annotation. (**D**) A novel MMDL hybrid architecture fused the extracted radiomics features and deep learning features for our multi-task prediction.

Here, we proposed a radiogenomics-based multi-label multi-task deep learning (MMDL) system to analyze 8-panel, 10-panel, and subtype expression simultaneously in a large-scale population (Figure 1). After experimenting with various technologies, a hybrid model that integrated radiomics and deep learning features achieved excellent performance that was readily aligned to clinical scenarios.

## 2. Methods

### 2.1. Study Population

**Figure 2:**
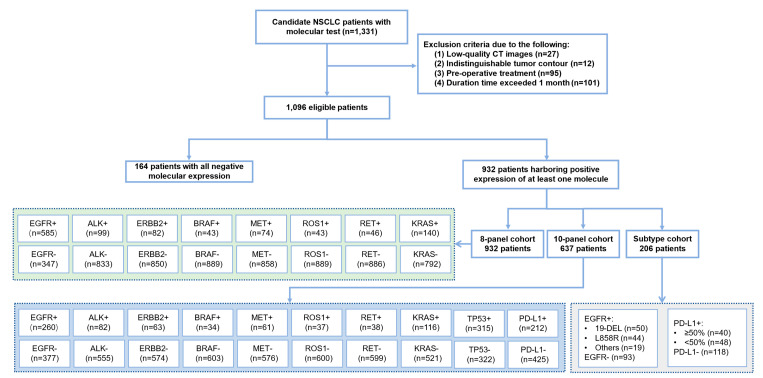
Illustration of cohort construction. Between April 2018 and June 2020, this study included a primary cohort of 1331 consecutive patients with NSCLC who visited West China Hospital of Sichuan University for model development and validation. Patients whose specimens underwent histological staining or were used for molecular testing (8-panel, 10-panel and subtype) were used to evaluate the performance of our models on binary classification of molecular status (positive and negative), prediction of multiple molecular alterations and classification of subtype. ‘+’ indicates positive type while ‘-’ means negative type.

The data for all NSCLC patients who visited West China Hospital of Sichuan University from April 2018 to June 2020 were collected in this study (Figure 2). Complete anonymization of data was performed before inclusion. Patients who met the following inclusion criteria were enrolled in this study: (1) histologically diagnosed with NSCLC; (2) had molecular tests including Amplification Refractory Mutation System-Polymerase Chain Reaction (ARMS-PCR) or NGS to confirm the status of *EGFR*, *ALK*, *ERBB2*, *BRAF*, *MET*, *ROS1*, *RET*, *KRAS* (8-panel), and *TP53*; PD-L1 expression status was detected using the SP142 antibody in IHC assays performed on the Ventana Benchmark platform; and (3) had a preoperative CT examination performed within 1 month before diagnosis. 

Patients were excluded from the study based on the following criteria: (1) low-quality CT images with image artifacts (due to metal objects) or motion artifacts (including breathing); (2) indistinguishable tumor contour that was unsuitable for CT segmentation due to nearby obstructive pneumonia and atelectasis; and (3) preoperative treatment had been received. Finally, on the basis of the aforementioned criteria, 1096 patients were identified with a diagnosis of NSCLC and definite multiple molecular expression status (positive and negative type); 932 patients were chosen to form the 8-panel cohort; 637 patients were chosen to form a 10-panel cohort (8 genes plus *TP53* and PD-L1) for further prediction, and 206 patients were collected for subtype prediction.

### 2.2. Imaging Acquisition and Preprocessing

We retrieved DICOM files of the CT scan from the Picture Archiving and Communication System (PACS). All scans had a reconstructed slice thickness ranging from 1 mm to 5 mm, a voltage of 120 kV, a current of 200–350 mA, and a matrix size of 512 × 512. CT scans typically store raw voxel intensities in Hounsfield units (HU), and the raw voxel value was normalized to 0 to 255 with a windowing based on the lung window.

To train the radiomics and deep learning models, we needed to acquire the delineation of the mutation-related nodules in advance. Given that manually segmenting the contours of chest abnormalities according to the original records might be time-consuming, it was vital to leverage automated contour extraction approaches to produce large-scale annotated molecular datasets. Automated AI segmentation models can be employed to automatically delineate all the lung nodules in a CT scan. However, sometimes there might be more than one nodule in a CT scan. In this instance, clinicians were needed to identify the targeted nodule manually. Therefore, the whole mask generation process required a two-phase procedure.

First, we adopted an off-the-shelf DenseNet as model backbone to automatically segment all nodule areas in the chest CT images [29]. The lesion segmentation model employed a feature pyramid block to form the U-shaped architecture, which is widely used to build segmentation models [30]. Then, radiologists with at least 5 years of expertise in thoracic diseases diagnosis could quickly review the final segmentation results and localize the targeted nodules according to previous inspection reports to form the final datasets. 

As a further pre-processing step, nodule regions of interests (ROIs) and mask ROIs were first cropped based on the confirmed nodule mask and then normalized to a size of 64×64×64 using third-order spline interpolation for further analysis. The training set was then balanced using data augmentation techniques such as horizontal flipping, random rotation, random blurring, and reweighting.

### 2.3. Radiomics Approach

To automatically extract radiomics features from CT scans, the radiomics approach employed an open-source Python package called Pyradiomics (version 3.0.1). To ensure data point validity, a total of 1052 radiomics features were extracted from each ROI; we considered only the 9 largest metastatic sites in each lesion, which are comprised of 19 first-order features, 16 shape features (3D), 10 shape features (2D), 24 Gray Level Co-occurrence Matrix (GLCM) features, 16 Gray Level Size Zone Matrix (GLSZM) features, 16 Gray Level Run Length Matrix (GLRLM) features, 5 Neighbouring Gray Tone Difference Matrix (NGTDM) features, and 14 Gray Level Dependence Matrix (GLDM) features. These features were also subdivided according to the image types (original image, LoG filter image and eight wavelet decomposition images).

Due to the high dimensionality of the radiomics feature space, we analyzed the similarity of each feature pair to eliminate irrelevant or highly correlated features to improve the generalization ability and optimize the model. As a result, we started by removing features with a training-set variance less than 0.8. Next, we standardized all of the radiomics features by scaling each feature to a certain range in order to keep features with a 2-norm value. The K-best feature selection method was then used for the normalized radiomics features, and the remaining features were applied to the least absolute shrinkage and selection operator (LASSO) penalized Cox proportional hazards regression method. The customized signature was then created by combining all critical features in a weighted linear fashion, and the personalized signature score was calculated for each lesion.

### 2.4. Convolutional Neural Network-Based Deep Learning

**Figure 3:**
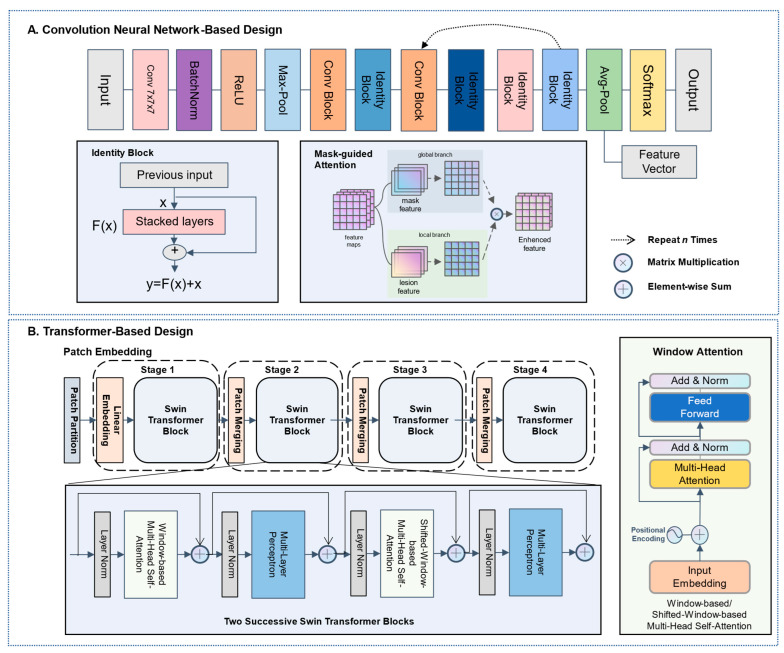
Overall framework of the proposed CNN-based and transformer-based network. Both proposed networks took two patches as inputs: the standardized lesion-ROI and associated mask-ROI combined two-channel volume. Before the first stage of each backbone, a mask-guide mechanism was employed to boost the model’s representation capacity. (**A**) The ResNet-3D network was developed by applying a ResNet3D-18 feature extractor to each 3D volume and employing multiple binary cross-entropy loss functions. (**B**) The Transformer network relied on the 3D-Swin-transformer as the backbone. The 3D-Swin-transformer merged image patches to build hierarchical feature maps. Two Successive Swin Transformer Blocks performed cyclic shift of local windows for shifted-window-based self-attention computation and the multi-head self-attention Module computes self-attention within each local 3D window.

The convolutional neural network (CNN)-based design relied on the ResNet-3D as a backbone with a lesion mask-guided attention mechanism to focus on lesion regions, enhancing lesion response while suppressing others [31]. We applied the lesion mask-guided attention to mine the lesion-mask enhanced feature and pay more attention to the interaction between lesions and surrounding tissues, thereby increasing the model’s representation capacity (Figure 3A, mask-guided attention). First, the standardized lesion-ROI and associated mask were separated into two images, which were then fed into the convolutional layer to obtain deep features of the lesion and surrounding tissues. Second, the similarity between the lesion and tissue pixels was determined, and the similarity was then normalized to obtain the weight of each point, which was then multiplied by the features of the corresponding point mapping. This method took into account the detailed information of the focus region, its distribution position in the whole image, and the concurrent reliance of other neighboring areas. The greater the similarity degree and effect on the point, the more other points are connected to this point. This mask-guide mechanism was employed at the beginning of the backbone. Then, several identity blocks were used to allow information to flow more smoothly from one layer to the next layer (Figure 3A, identity block). Finally, global average pooling was used to replace the model’s top layers, after which a fully connected layer of 512 nodes (dimensions of the deep learning features) with rectified linear unit (ReLU) activation functions and a fully completely connected layer with 8 or 10 nodes (8-panel and 10-panel) were created with the Softmax activation function.

### 2.5. Transformer-Based Deep Learning

The main architecture of the 3D-Swin-transformer comprised four stages, with each level reducing the resolution of the input feature map and expanding the receptive field layer by layer, similar to the CNN. The model mainly consisted of three components (Figure 3B): (1) patch embedding: for the input 3D ROI, linear embedding changes the dimensions of the input vector to preset tokens that can be processed by the Transformer; (2) patch merging: the function of this module was to perform down-sampling before the start of each stage, which was used to reduce the resolution and adjust the number of channels to form a hierarchical design; the 3D-Swin-transformer could generate hierarchical feature maps at various resolutions by patch merging layers, making it suitable as a general-purpose backbone for pixel-level operations; and (3) window attention: this calculated the relationships between each patch in an ROI and all the patches in the ROI, and to a certain extent, the relationship between these patches reflected the relevance and importance of the different patches in the ROI. The attentional mechanism, of which the self-attention was the core of the coding unit, and the most significant component of the proposed transformer-based paradigm at each transformer block. The window-based multi-head self-attention and shifted-window-based multi-head self-attention were successively applied in each Swin-transformer block to further extract global interactions between adjacent window patches (Figure 3B, two successive Swin transformer blocks). Notably, to emphasize the nodule areas when extracting the deep features, we also adopted the two-channel input for the transformer model to achieve lesion mask-guided feature extraction.

### 2.6. Multi-Label Multi-Task Deep Learning (MMDL) System

For each task, the multi-label multi-task deep learning (MMDL) system achieved multi-label prediction by using multiple binary classifiers to analyze whether patients have positive expression of molecules such as *EGFR*, *ALK*, *ERBB2*, etc. In order to achieve the multiple tasks, the model employed full connectivity to allow for self-adaptation based on a combination of deep learning and radiomics features (Figure 1D). Due to these related features originate in various dimension spaces, how to integrate these features to develop joint models was currently not addressed. In this study, we first used different approaches (radiomics-based and transformer-based) to extract the radiomics features and deep-learning features. In order to investigate a better feature fusion, we computed the correlation heatmap of radiomics and deep learning features to visualize the relative contributions of features on molecular status prediction. As a result, the reference distribution was the correlation distribution across all features from the patient case. The XGboost approach was then used to select the key features identified as the most significant contributors, which were followed by a new buffer layer, to an embedding feature space, making superior use of the individual feature strength [32]. Finally, various features were embedded to complete multiple tasks such as gene mutation analysis, molecular expression analysis, and subtype identification.

### 2.7. Statistical Analysis

The performances of the models were assessed according to AUC, specificity, and sensitivity with a 95% CI. To generate averages and standard deviations for each set of cross-validation trials, performance indicators were averaged over k folds. Moreover, the cut-point was determined by maximizing the sum of sensitivity and specificity, which was similar to selecting a point on the receiver operating characteristic curve (ROC). For each genetic mutation, DeLong’s test was used to assess the diagnostic performance of the radiomics model, deep learning model, and combined model. All of the statistical tests were two-sided, with *p* < 0.05 denoting statistical significance.

For feature selection, model creation, and performance evaluation, the scikit-learn package (Python v3.8, Scikit-learn v0.24, https://scikit-learn.org, accessed on 1 September 2022) was utilized. Pyradiomics software (version 3.0.1) was used for feature extraction. The PyTorch (version 1.5.1) and torchvision (version 0.7.1) packages were utilized to extract deep learning features.

## 3. Results

### 3.1. Patient Characteristics

We established a Cancer Shared Database (CSD), covering radiology images and molecular information of 1096 patients diagnosed with NSCLC at West China Hospital of Sichuan University (Figure 1A). The CSD was divided into four cohorts according to the different prediction tasks (Figure 1B): a binary expression cohort (positive expression of at least one molecule and all negative molecular expression, *n* = 1096, 58.26 ± 10.69 years old), an 8-panel cohort (8 gene mutations analysis, *n* = 932, 58.11 ± 10.64 years old), a 10-panel cohort (10 molecular status analysis, *n* = 637, 58.26 ± 11.03 years old), and a subtype identification cohort (*EGFR* subtypes and PD-L1 expression, *n* = 206) (Table S1). The NGS test identified 585 (62.8%), 99 (10.6%), 82 (8.8%), 43 (4.6%), 74 (7.9%), 43 (4.6%), 46 (4.9%), and 140 (15.0%) patients who had *EGFR*, *ALK*, *ERBB2*, *BRAF*, *MET*, *ROS1*, *RET*, and *KRAS* mutations (8-panel), respectively (Figure 2). Among the *EGFR*-mutant patients, 50 (44.2%) harbored an exon 19-DEL, 44 (38.9%) displayed an exon 21 L858R, and finally 19 (16.8%) cases showed a rare *EGFR* mutation. Among PD-L1 positive expression patients, 40 patients had high PD-L1 expression (TPS ≥ 50%), whereas 48 patients had low PD-L1 expression (TPS ≥ 1% and <50%). 93 patients harbored wild-type *EGFR*, and 118 patients had negative expression of PD-L1.

In the image preprocessing process, the DenseNet model with feature pyramid networks was utilized to automatically segment all lesion areas in chest CT images (Figure 1C). Then radiologists with at least 5 years of expertise in thoracic tumor diagnosis quickly reviewed the segmentation results to form the training and validation datasets. Afterward, the cropped nodule ROIs were standardized to the same size for model construction. Furthermore, we explored various AI approaches and an improved combination of methods to evaluate the association between the features extracted using a standard radiomics pipeline and those extracted using the deep learning pipeline (CNN-based or transformer-based). Finally, the MMDL system which integrated 512 deep learning features and 20 highest-performing radiomics features was established to achieve the simultaneous prediction of multiple molecular statuses (Figure 1D). For each task cohort, we randomly split the dataset into training (80%), validation (10%), and testing (10%) subsets.

### 3.2. The Performance of the Radiomics Model

Radiomics, a classic machine learning method, initially identified 1052 relevant features from the ROI of each patient. According to Bonferroni correction, 512 radiomics features were chosen and then reduced to 20 possible predictors using LASSO regression. The target molecular expression classification resulted in AUCs of 0.818 (95% CI, 0.773–0.871), and 0.807 (95% CI, 0.738–0.884) in the validation cohort and testing cohort, respectively, with a sensitivity and specificity of 0.680 (95% CI, 0.629–0.739) and 0.840 (95% CI, 0.758–0.912) for the validation cohort and 0.856 (95% CI, 0.805–0.904) and 0.722 (95% CI, 0.607–0.853) for the testing cohort (Table 1). Regarding the 8-panel classification task, the AUCs of the radiomics model (Figure S1) for discriminating *EGFR*, *ALK*, *ERBB2*, *BRAF*, *MET*, *ROS1*, *RET* and *KRAS* were 0.796 (95% CI, 0.783–0.857), 0.867 (95% CI, 0.746–0.971), 0.757 (95% CI, 0.654–0.876), 0.680 (95% CI, 0.489–0.911), 0.915 (95% CI, 0.838–0.985), 0.822 (95% CI, 0.683–0.961), 0.816 (95% CI, 0.669–0.936) and 0.818 (95% CI, 0.716–0.920), respectively. The accuracy of the predictive model for the 8-panel was 95.1% in the validation dataset and 92.8% in the testing dataset, suggesting that this model was not prone to making errors and implicitly learned the relationship among these categories. Similar results were also obtained in terms of the 10-panel cohort and the subtype cohort.

### 3.3. The Performance of the Deep Learning Models

The performance of deep learning models was generally superior to those of the radiomics model, regardless of whether the CNN-based model or transformer-based model was used (Table 2). The transformer-based design relied on the 3D-Swin-transformer as the backbone along with a lesion mask-guided feature extraction scheme [33]. The model first employed the patch partition operation to separate the standardized lesion-ROI and related mask combined two-channel volume into tiny patches, which were then fed into the shifted window transformer block, to model the long-range decencies among and within those tiny patches. The quantitative performance was shown in Table 2 and the detailed diagnostic measures of all CNN-based and transformer-based models were shown in Figures S2 and S3, indicating that the transformer-based model gained better performance than the CNN-based model with a significant difference (AUC = 0.847, 95% CI, 0.763–0.942 versus AUC = 0.825, 95% CI, 0.682–0.891 in the target molecular testing cohort, *p* < 0.0001). A similar improvement over the CNN-based model was also observed in the other cohorts, supporting the idea that the transformer-based features can be selected for deep learning prediction models that can automatically extract better deep features of the ROI to predict the molecular expression.

### 3.4. Performance of the Proposed MMDL Hybrid Model

**Figure 4:**
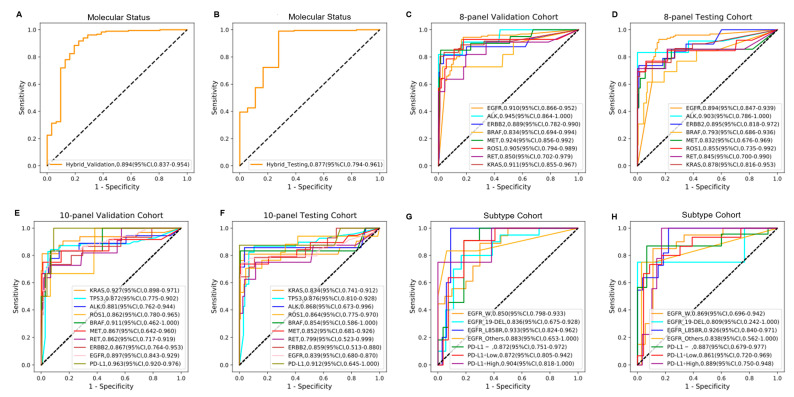
MMDL hybrid model performances in the prediction of multiple molecular alterations. (**A**,**B**) The ROC curves for predicting target molecular status (positive and negative) in the validation set and testing set, respectively. (**C**,**D**) The ROC curves for predicting multiple mutations in the 8-panel cohort in the validation set and testing set, respectively. (**E**,**F**) The ROC curves for predicting multiple alterations in the 10-panel cohort in the validation set and testing set, respectively. (**G**,**H**) The ROC curves for predicting molecular expression in the subtype cohort in the validation set and testing set, respectively, *EGFR*_W and PD-L1- represent wild type of *EGFR* and negative expression of PD-L1. PD-L1+ was separated into PD-L1+ Low and PD-L1+ High according to the TPS cutoff of 50%.

In all sessions, the MMDL hybrid model based on the integration radiomics and deep learning features achieved the best discriminative performance (Table 3, Figure 4). The AUCs of the hybrid model in the validation and testing sets yielded 0.894 (95% CI, 0.837–0.954) and 0.877 (95% CI, 0.794–0.961) for binary prediction, 0.896 (95% CI, 0.802–0.983) and 0.862 (95% CI, 0.758–0.969) for 8-panel identification, 0.891 (95% CI, 0.756–0.952) and 0.856 (95% CI, 0.663–0.948) for 10-pannel molecular status assessment, and 0.879 (95% CI, 0.761–0.962) and 0.868 (95% CI, 0.641–0.972) for *EGFR* and PD-L1 subtype classification, respectively. In the eight-gene prediction task, the AUC of each specific gene ranged from 0.793 to 0.903. For the ten-molecules evaluation task, the prediction performance for the original eight genes fluctuated, but *TP53* and PD-L1 could be successfully predicted with AUCs of 0.876 (95% CI, 0.810–0.928) and 0.912 (95% CI, 0.645–1.000), respectively. Moreover, the model showed a relatively stable sensitivity and specificity of 0.990(0.979–1.000) and 0.722(0.550–0.905) for target alterations, 0.759 (95% CI, 0.591–0.933) and 0.948 (95% CI, 0.922–0.973) for discriminating the 8-panel cohort, 0.797 (95% CI, 0.623–0.947) and 0.953 (95% CI, 0.929–0.975) for the 10-panel cohort, and 0.850 (95% CI, 0.642–0.977) and 0.902(95% CI, 0.794–0.976) for the subtype cohort, respectively.

Compared to that of single-feature models, the performance of the fusion model was improved. For example, compared with the transformer-based deep learning model, the hybrid model significantly improved the AUC of discriminating *TP53* mutation from 0.834 (95% CI, 0.757–0.891) to 0.876 (95% CI, 0.810–0.928). In addition to genotype mining, the hybrid model also had excellent potential for subtype analysis (AUC = 0.868, 95% CI, 0.641–0.972) compared with that of the deep learning model (AUC = 0.843, 95% CI, 0.718–0.924) and the radiomics model (AUC = 0.732, 95% CI, 0.536–0.925), indicating that the presence of a mutation correlates with both semantic information (deep learning features) and the texture information (radiomics features).

### 3.5. Correlation Analysis between Radiomics and Deep Learning Features

To further illustrate the association between deep learning and radiomics features in predicting multiple molecular alterations and mutation status, we utilized a variety of methodologies to develop a better fusion expression of tumor characteristics. Within each feature bank, we employed 20 radiomics and 512 transformer-based features to produce a heatmap that depicted the correlation between the two feature sets. In Figure S4A, each dot represented a correlation coefficient, and the red color meant a coefficient of zero, whereas the white and black dots reflect positive and negative correlations, respectively. The heatmap demonstrated a strong linear relationship between important features (radiomics vs. deep learning features). Figure S4B depicted the predictive performance of radiomics and deep learning features for positive and negative patients, with practically all CT volume characteristics demonstrating a strong ability to differentiate between the two groups.

**Figure 5:**
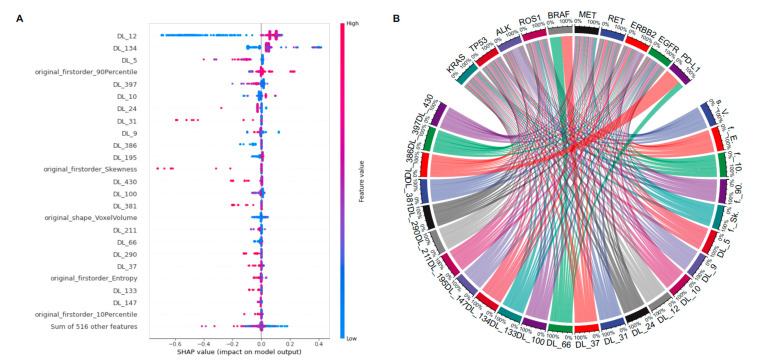
Visualization of the identified radiomics and deep learning features. (**A**) Multivariable logistic regression to identify radiomics and deep learning features associated with NSCLC patients. Abbreviations: DL, deep learning. (**B**) The correlations between selected features and ten molecules. The width of the link indicated the relative strength.

However, because these related features originated in separate feature dimension spaces, the usual feature selection approach could not be simply applied. As a result, we applied the SHapley Additive exPlanation (SHAP)-based XGboost method to complete the multivariable logistic regression and calculate the influence of a given variable on a given feature in contrast to the prediction (Figure 5A) [34]. Furthermore, 19 deep learning and 5 radiomics key features were significantly associated with more than one molecule, indicating the potential of fusion features in predicting molecular co-alteration status. (Figure 5B).

## 4. Discussion

Radiogenomic approaches aggregate radiology and genomics data based on the hypothesis that radiomic features reflect macroscopic and molecular properties of tissues. Such tests in routine imaging could offer the ability to capture features from a full 3D volume of the tumor, avoiding sampling errors due to intra-tumor heterogeneity. In this research, a hybrid deep learning model named MMDL was developed to evaluate actionable mutations and PD-L1 expression non-invasively based on CT images of 1096 patients with lung cancer. This approach combined 512 transformer-based deep learning features and approximately 20 radiomic features to predict 10 molecular states with AUC performance above 0.799 (Figure 4C).

To the best of our knowledge, this is the first study to predict mutations in 8 actionable genes or even 10 molecules based on CT images. The predictive performance of the MMDL model was excellent in identifying distant molecular and subtype status, which has potential for clinical application. It could aid in the assessment of patients’ molecular status non-invasively and assist clinicians in making diagnosis and treatment decisions. However, the predictive performance varied among different molecules. For example, the best AUC of the MMDL model in the 8-panel task was 0.903 (95% CI, 0.786–1.000) for *ALK* and the worst AUC was 0.793 (95% CI, 0.686–0.936) for *BRAF*. This may have been related to different gene frequencies and training sample sizes (82 patients with *ALK* mutation; 34 patients with *BRAF* mutation). The same situation occurred in the prediction of genetic mutations using pathological images. Some investigators developed a CNN model based on Inception v3 architecture for the automatic analysis of tumor slides using publicly available whole-slide images available in the TCGA [35]. They found that six mutated genes which were mutated in at least 10% of the available tumors, including *STK11*, *EGFR*, *FAT1*, *SETBP1*, *KRAS, and TP53*, could be predicted from pathology images with AUCs ranging from 0.733 to 0.856, but their model was not able to detect genes with lower incidence, such as *ALK*. This suggested the urgent need for a publicly shared database to enable construction of more accurate artificial intelligence models.

In order to efficiently extract image features for molecular prediction, we established deep learning models based on transformer and CNN architectures, respectively. Transformer is a novel deep learning network that avoids recurrence and completely relies on the attention mechanism to model the global dependencies of the input and output. This model breaks through the limitation that the recurrent neural network (RNN) model cannot be calculated in parallel, and the number of operations required to calculate the association between two locations does not increase with distance compared to CNN. Furthermore, the MMDL model integrated radiomics and deep learning features to achieve remarkable prediction results. Different modalities of medical data provide patient diagnosis and treatment information from a specific perspective. The characteristics of clinical multimodal data provide the basis for the realization of accurate disease diagnosis [36,37]. Some researchers conducted Tumor Origin Assessment via Deep Learning (TOAD) to predict the origins of 18 common tumor primary/metastases and unknown primary cancer origins based on a multiclass, multitask, multiple-instance learning architecture. Compared with that of the single-modal single-task model, the performance of the fusion model was 2.0% higher in primary tumor prediction, and 6.8% higher in tumor metastasis prediction, and the overall accuracy rate reached 83.4% [38]. Multimodal data fusion is a future trend in the development of medical diagnosis and treatment methods.

There were some limitations in this study. First, all data came from a single medical center, so the generalization of the model requires multicenter data for verification. Second, the deep learning process was invisible and lack of interpretability. We have explored feature correlations, but there was still a certain distance to clinical practice. Third, this study focused on multiple molecular statuses and lacked patient efficacy and prognostic assessments. Previous studies have confirmed that deep learning features related to molecular status can be used to evaluate efficacy in patients [39], and we will conduct more in-depth and detailed research in the future.

## 5. Conclusions

The MMDL system was established and validated to achieve excellent predictive performance for 10 molecular alterations and specific subtypes in NSCLC. Radiogenomic model was a combination of routine clinical radiological scans and artificial intelligence to detect molecular status non-invasively. It was the potential decision-support tool to assist physicians in cancer treatment management.

## Figures and Tables

**Table 1:** Predictive performance of radiomics model.

Prediction Task	Dataset	Sensitivity (95%CI)	Specificity (95%CI)	Accuracy (95%CI)	AUC (95%CI)
Binary	Validation	0.680 (0.629–0.739)	0.840 (0.758–0.912)	0.836 (0.803–0.881)	0.818 (0.773–0.871)
	Testing	0.856 (0.805–0.904)	0.722 (0.607–0.853)	0.829 (0.789–0.874)	0.807 (0.738–0.884)
8-panel	Validation	0.814 (0.625–0.980)	0.833 (0.802–0.868)	0.951 (0.933–0.971)	0.831 (0.702–0.949)
	Testing	0.691 (0.504–0.888)	0.882 (0.839–0.921)	0.928 (0.894–0.959)	0.809 (0.692–0.927)
10-panel	Validation	0.796 (0.656–0.933)	0.852 (0.810–0.896)	0.901 (0.869–0.933)	0.847 (0.762–0.936)
	Testing	0.705 (0.496–0.918)	0.880 (0.836–0.918)	0.876 (0.836–0.915)	0.821 (0.703–0.936)
Subtype	Validation	0.820 (0.640–0.961)	0.769 (0.642–0.887)	0.754 (0.646–0.861)	0.771 (0.606–0.900)
	Testing	0.741 (0.443–0.968)	0.793 (0.654–0.914)	0.783 (0.682–0.894)	0.732 (0.536–0.925)

**Table 2:** Predictive performance of deep learning models.

Deep Learning Algorithm	Prediction Task	Dataset	Sensitivity (95% CI)	Specificity (95% CI)	Accuracy (95% CI)	AUC (95% CI)
CNN-Based	Binary	Validation	0.919 (0.879–0.955)	0.724 (0.621–0.857)	0.884 (0.854–0.933)	0.836 (0.777–0.911)
		Testing	0.960 (0.924–0.982)	0.611 (0.464–0.743)	0.611 (0.464–0.743)	0.825 (0.682–0.891)
	8-panel	Validation	0.767 (0.636–0.883)	0.906 (0.879–0.933)	0.943 (0.922–0.963)	0.869 (0.745–0.926)
		Testing	0.721 (0.588–0.864)	0.932 (0.907–0.954)	0.946 (0.926–0.966)	0.839 (0.757–0.931)
	10-panel	Validation	0.743 (0.592–0.902)	0.932 (0.905–0.956)	0.937 (0.914–0.960)	0.848 (0.732–0.921)
		Testing	0.706 (0.563–0.844)	0.906 (0.877–0.933)	0.924 (0.900–0.948)	0.829 (0.724–0.888)
	Subtype	Validation	0.858 (0.692–0.973)	0.830 (0.700–0.939)	0.840 (0.742–0.923)	0.839 (0.673–0.933)
		Testing	0.881 (0.765–0.972)	0.764 (0.622–0.885)	0.786 (0.684–0.884)	0.810 (0.648–0.915)
Transformer-Based	Binary	Validation	0.967 (0.943–0.984)	0.710 (0.579–0.840)	0.930 (0.906–0.953)	0.857 (0.782–0.931)
		Testing	0.979 (0.964–0.995)	0.632 (0.467–0.826)	0.944 (0.920–0.967)	0.847 (0.763–0.942)
	8-panel	Validation	0.758 (0.598–0.917)	0.962 (0.940–0.978)	0.950 (0.927–0.973)	0.872 (0.774–0.969)
		Testing	0.746 (0.573–0.926)	0.970 (0.951–0.987)	0.956 (0.936–0.978)	0.863 (0.752–0.968)
	10-panel	Validation	0.785 (0.597–0.947)	0.918 (0.886–0.948)	0.941 (0.913–0.965)	0.864 (0.743–0.935)
		Testing	0.733 (0.559–0.910)	0.925 (0.898–0.949)	0.941 (0.914–0.967)	0.842 (0.690–0.917)
	Subtype	Validation	0.749 (0.553–0.958)	0.941 (0.886–0.988)	0.883 (0.814–0.957)	0.855 (0.701–0.912)
		Testing	0.760 (0.592–0.924)	0.932 (0.877–0.975)	0.862 (0.796–0.936)	0.843 (0.718–0.924)

**Table 3:** Predictive performance of MMDL hybrid model.

Prediction Task	Dataset	Sensitivity (95% CI)	Specificity (95% CI)	Accuracy (95% CI)	AUC (95% CI)
Binary	Validation	0.918 (0.891–0.952)	0.774 (0.667–0.903)	0.930 (0.906–0.958)	0.894 (0.837–0.954)
	Testing	0.990 (0.979–1.000)	0.722 (0.550–0.905)	0.962 (0.939–0.986)	0.877 (0.794–0.961)
8-panel	Validation	0.829 (0.669–0.986)	0.927 (0.900–0.955)	0.956 (0.934–0.978)	0.896 (0.802–0.983)
	Testing	0.759 (0.591–0.933)	0.948 (0.922–0.973)	0.954 (0.933–0.977)	0.862 (0.758–0.969)
10-panel	Validation	0.827 (0.678–0.945)	0.914 (0.881–0.947)	0.948 (0.923–0.972)	0.891 (0.756–0.952)
	Testing	0.797 (0.623–0.947)	0.953 (0.929–0.975)	0.953 (0.928–0.976)	0.856 (0.663–0.948)
Subtype	Validation	0.870 (0.689–0.987)	0.858 (0.761–0.952)	0.842 (0.748–0.921)	0.879 (0.761–0.962)
	Testing	0.850 (0.642–0.977)	0.902 (0.794–0.976)	0.876 (0.778–0.951)	0.868 (0.641–0.972)

## Data Availability

The data presented in this study are available on request from the corresponding author.

## Supplementary Materials

The following supporting information can be downloaded at: https://www.mdpi.com/article/10.3390/cancers14194823/s1, Figure S1: The radiomics model performance in the prediction of multiple molecular alterations; Figure S2: The CNN-based deep learning model performance in the prediction of multiple molecular alterations; Figure S3: The transformer-based deep learning model performance in the prediction of multiple molecular alterations; Figure S4: Association between radiomics and deep learning features; Table S1: The clinical characteristic of cancer shared dataset.
