# Supplementary material for: Radiogenomic System for Non-Invasive Identification of Multiple Actionable Mutations and PD-L1 Expression in Non-Small Cell Lung Cancer Based on CT Images
Source: Cancers (Basel). 2022 Oct 2;14(19):4823. doi: 10.3390/cancers14194823 (PMC9563625; doi:10.3390/cancers14194823)
Supplement: Supplementary file 1 [file cancers-14-04823-s001.zip › cancers-1931869-supplementary.pdf]

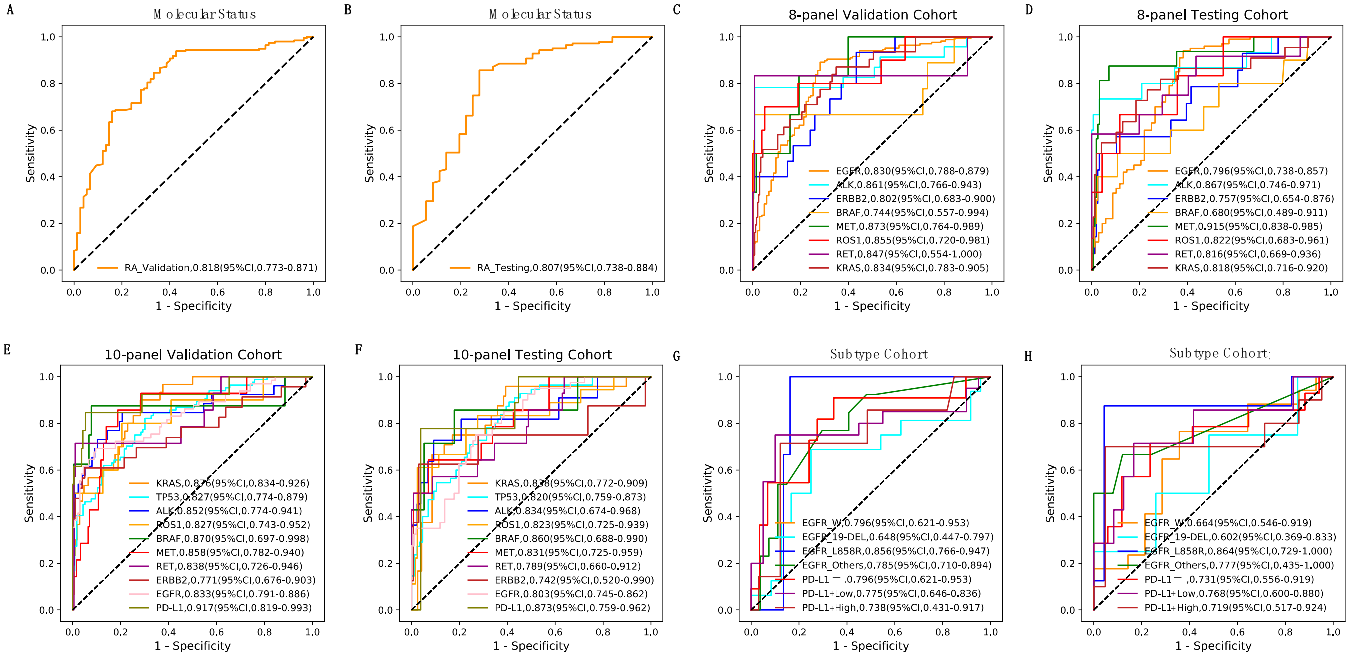

Figure S1. The radiomics model performances in the prediction of multiple molecular alterations. (**A**,**B**) The ROC curves for predicting target molecular status (positive-type and negative-type) in the validation set and testing set, respectively. (**C**,**D**) The ROC curves for predicting multiple mutations in the 8-panel cohort in the validation set and testing set, respectively. (**E**,**F**) The ROC curves for predicting multiple alterations in the 10-panel cohort in the validation set and testing set, respectively. (**G**,**H**) The ROC curves for predicting molecular ex-pression in the subtype cohort in the validation set and testing set, respectively, EGFR_W and PD-L1- represent wild-type of EGFR and negative expression of PD-L1. PD-L1+ was separated into PD-L1+ Low and PD-L1+ High according to the TPS cutoff of 50%.

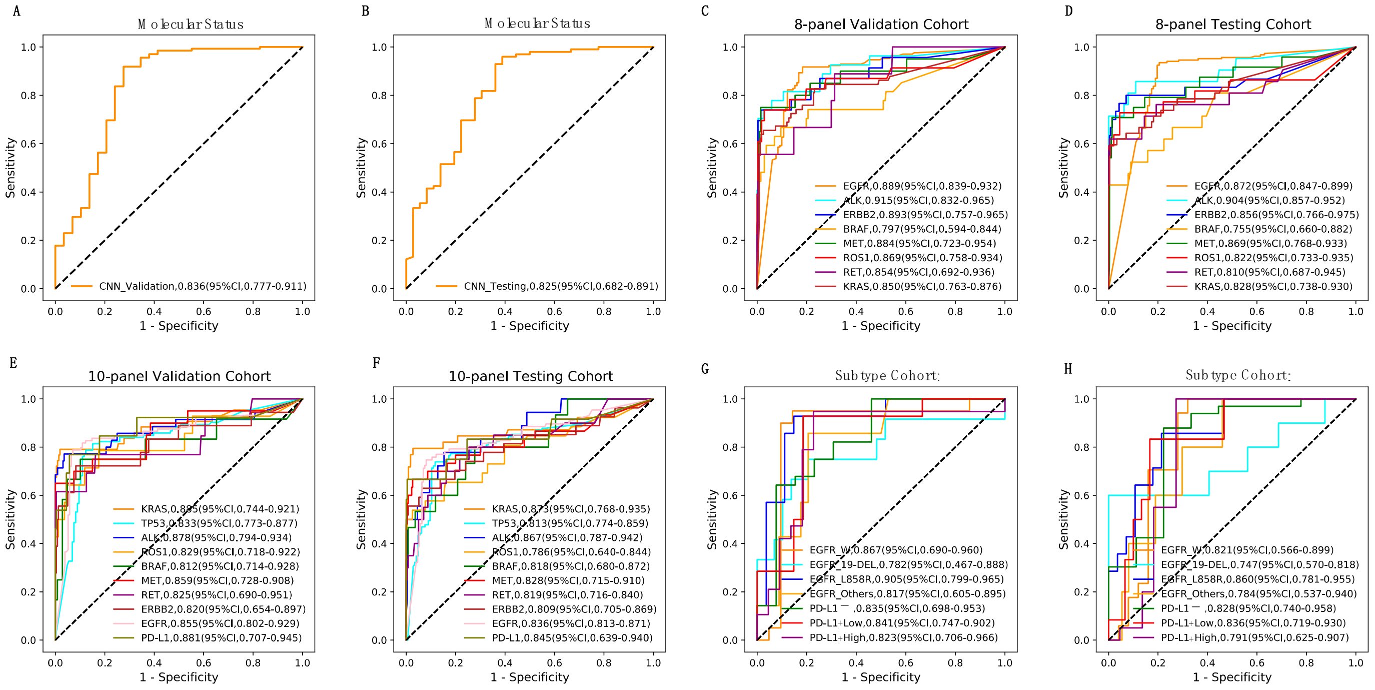

Figure S2. The CNN-based deep learning model performances in the prediction of multiple molecular alterations. (**A**,**B**) The ROC curves for predicting target molecular status (positive-type and negative-type) in the validation set and testing set, respectively. (**C**,**D**) The ROC curves for predicting multiple mutations in the 8-panel cohort in the validation set and testing set, respectively. (**E**,**F**) The ROC curves for predicting multiple alterations in the 10-panel cohort in the validation set and testing set, respectively. (**G**,**H**) The ROC curves for predicting molecular ex-pression in the subtype cohort in the validation set and testing set, respectively, EGFR_W and PD-L1- represent wild-type of EGFR and negative expression of PD-L1. PD-L1+ was separated into PD-L1+ Low and PD-L1+ High according to the TPS cutoff of 50%.

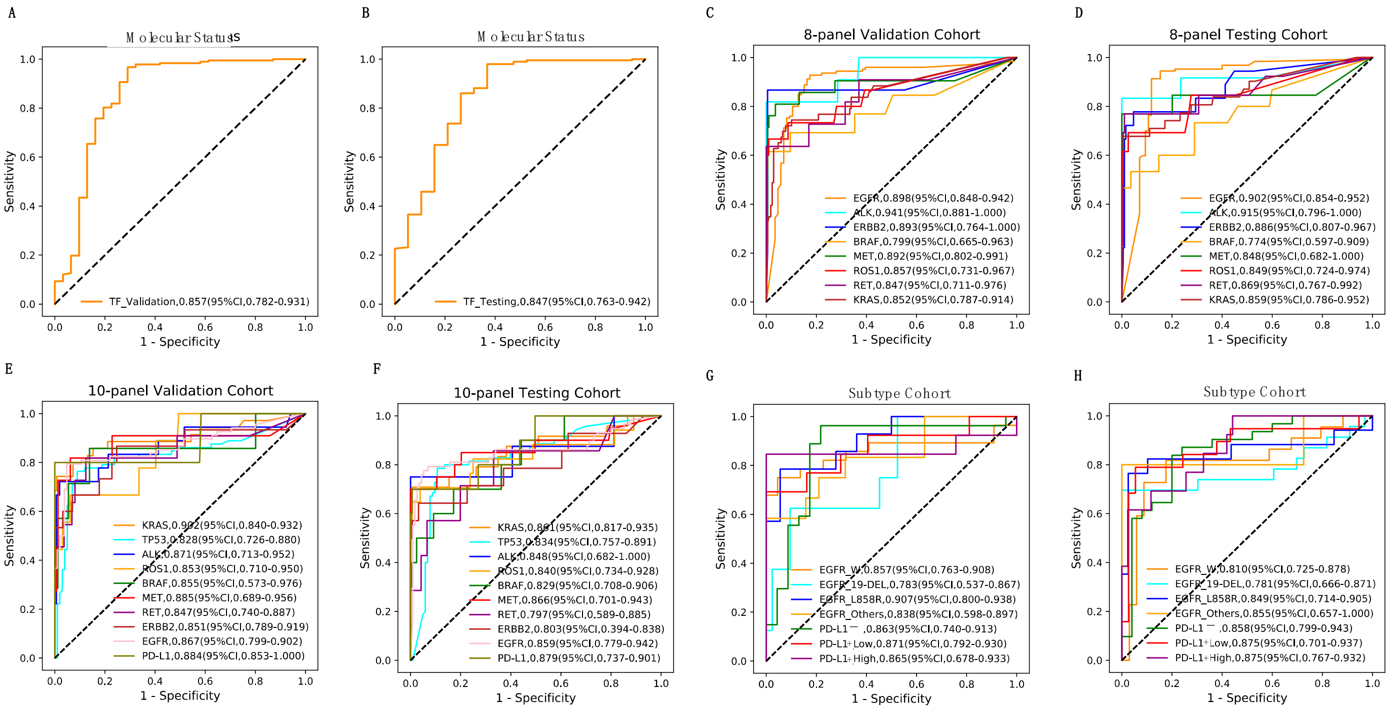

Figure S3. The transformer-based deep learning model performances in the prediction of multiple molecular alterations. (**A**,**B**) The ROC curves for predicting target molecular status (positive-type and negative-type) in the validation set and testing set, respectively. (**C**,**D**) The ROC curves for predicting multiple mutations in the 8-panel cohort in the validation set and testing set, respectively. (**E**,**F**) The ROC curves for predicting multiple alterations in the 10-panel cohort in the validation set and testing set, respectively. (**G**,**H**) The ROC curves for predicting molecular ex-pression in the subtype cohort in the validation set and testing set, respectively, EGFR_W and PD-L1- represent wild-type of EGFR and negative expression of PD-L1. PD-L1+ was separated into PD-L1+ Low and PD-L1+ High according to the TPS cutoff of 50%.

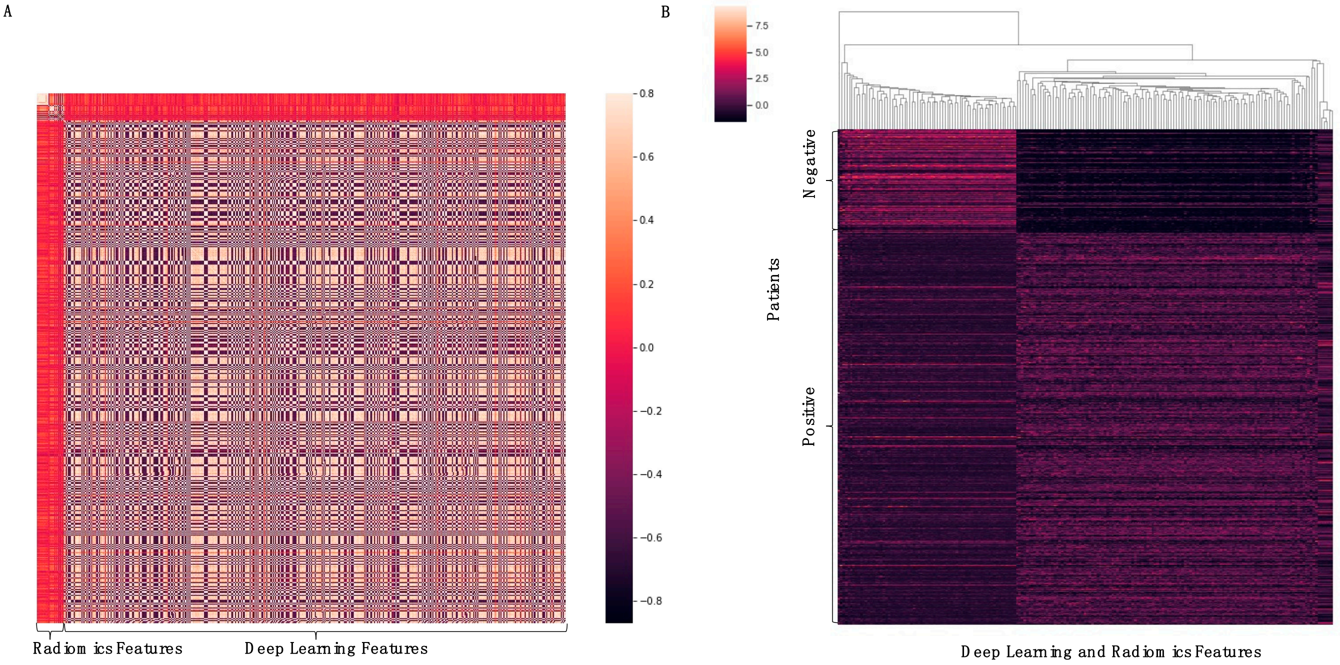

Figure S4. Association between radiomics and deep learning features. (**A**) Correlation heatmap of radiomics and deep learning features. (**B**) The correlation between selected features and the mutant genotypes patients in the development cohorts.

Table S1. The clinical characteristic of cancer shared dataset.

	All Patients<br>N = 1096	8-Panel Cohort<br>N = 932	10-Panel Cohort<br>N = 637
**Age (SD)**	58.26 (10.69)	58.11 (10.64)	58.26 (11.03)
**Sex**			
**Male**	598(54.6%)	481(51.6%)	393(61.7%)
**Female**	498(45.4%)	451(48.4%)	244(38.3%)
**Smoking history**			
**Current or former**	441(40.2%)	346(37.1%)	315(49.5%)
**Never**	581(53.0%)	525(56.3%)	317(49.8%)
**Unknow**	74(6.8%)	61(6.5%)	5(0.8%)
**Histology type**			
**LUAD**	875(79.8%)	786(84.3%)	517(81.2%)
**LUSC**	96(8.8%)	48(5.2%)	73(11.5%)
**Others**	125(11.4%)	98(10.5%)	47(7.4%)
**Actional mutation**			
EGFR			
**Mutant**	585(53.4%)	585(62.8%)	260(40.8%)
**Wild**	511(46.6%)	347(37.2%)	377(59.2%)
ALK			
**Mutant**	99(9.0%)	99(10.6%)	82(12.9%)
**Wild**	997(91.0%)	833(89.4%)	555(87.1%)
ERBB2			
**Mutant**	82(7.5%)	82(8.8%)	63(9.9%)
**Wild**	1014(92.5%)	850(91.2%)	574(90.1%)
BRAF			
**Mutant**	43(3.9%)	43(4.6%)	34(5.3%)
**Wild**	1053(96.1%)	889(95.4%)	603(94.7%)

MET			
**Mutant**	74(6.8%)	74(7.9%)	61(9.6%)
**Wild**	1022(93.2%)	858(92.1%)	576(90.4%)
ROS1			
**Mutant**	43(3.9%)	43(4.6%)	37(5.8%)
**Wild**	1053(96.1%)	889(95.4%)	600(94.2%)
RET			
**Mutant**	46(4.2%)	46(4.9%)	38(6.0%)
**Wild**	1050(95.8%)	886(95.1%)	599(94.0%)
KRAS			
**Mutant**	140(12.8%)	140(15.0%)	116(18.2%)
**Wild**	956(87.2%)	792(85.0%)	521(81.8%)
TP53			
**Mutant**	315(28.7%)	-	315(49.5%)
**Wild**	322(29.4%)	-	322(50.5%)
**Unknown**	459(41.9%)	-	-
**PD-L1 Expression**			
**Positive**	335 (30.6%)	-	212(33.3%)
**Negative**	432(39.4%)	-	425(66.7%)
**Unknown**	329(30.0%)	-	-

Abbreviations: LUAD, lung adenocarcinoma; LUSC, lung squamous cell carcinoma.
